# Strengthening Communities’ Youth Access Policies May Facilitate Clean Indoor Air Action

**Published:** 2007-09-15

**Authors:** Leonard A Jason, Yvonne M Hunt, Monica L Adams, Steven B Pokorny, Praveena B Gadiraju

**Affiliations:** Center for Community Research, DePaul University, Chicago, Illinois; Center for Community Research, DePaul University, Chicago, Illinois; Center for Community Research, DePaul University, Chicago, Illinois; Department of Health Education and Behavior, University of Florida, Gainesville, Florida; Center for Community Research, DePaul University, Chicago, Illinois

## To the Editor:

Reducing youth access to tobacco products has been advocated as one public health strategy to address the problem of youth tobacco use (1,2). Enactment and enforcement of laws prohibiting the possession, use, and purchase of tobacco by minors represent one approach to restricting youth tobacco access and decreasing public smoking among youth. Youth possession, use, and purchase (PUP) laws currently exist in 45 states (3); however, controversy exists on the appropriateness of continuing to direct tobacco control resources toward enforcing these laws. Although many community members and law enforcement officials endorse the use of PUP laws as a method for decreasing public smoking by youth, some anti-tobacco advocates are opposed to this public health policy tool. Opponents of PUP laws argue that the laws are conceptually flawed and difficult to enforce and unduly punish youth instead of placing responsibility on tobacco companies (4). In addition, PUP law critics have argued that investing more resources in communities' efforts to enforce PUP laws may divert attention from other forms of tobacco control (e.g., clean indoor air legislation) (5). Unfortunately, these criticisms have been made without supportive empirical data. For the first time, data from a recent randomized trial that involved implementing youth PUP law enforcement initiatives allow us to evaluate one of the criticisms of youth access policies.

In 2001, we randomly assigned 24 Illinois communities to either a control or an intervention group and then followed the 24 communities for 4 years. The 12 intervention communities agreed to initiate or increase PUP law enforcement practices, whereas the 12 control towns received instructions to maintain their current low levels of PUP law enforcement. The DePaul University Institutional Review Board approved of the study's design, including continuation of low levels of PUP law enforcement in the 12 control communities. Because the evidence on whether or not PUP law enforcement was effective in reducing youth smoking was still unclear, the IRB allowed us to experimentally evaluate this issue. All 24 towns had merchant enforcements to reduce illegal sales of tobacco.

The Table provides data on demographics, the mean number of PUP law citations issued annually, and the level of police readiness for each participating community. Control and intervention towns did not differ significantly at baseline in socioeconomic status, as measured by median household income and high school educational attainment, nor did they differ in race or ethnicity. A measure of the level of police department readiness to carry out tasks related to the enforcement of PUP laws (6) at baseline revealed no group differences. Although the study did not collect data on the level of community readiness to implement smoke-free ordinances (e.g., evidence of prior attempts to enact legislation), to our knowledge, no efforts to pass smoke-free ordinances were under way in these towns at baseline because the state had exclusive regulatory authority over public smoking. During the study, the mean number of PUP law citations issued to minors within the intervention communities was significantly higher than within the control communities, suggesting that PUP law enforcement efforts were stronger in these towns. We neither encouraged nor discouraged efforts on environmental tobacco smoke legislation in either intervention or control communities.

In 2001, all communities in Illinois operated under the same set of weak state regulations on environmental tobacco smoke, requiring only that public establishments, excluding bars, have a designated nonsmoking area. However, an amendment to the Illinois Clean Indoor Air Act in January 2006 granted regulatory authority over public smoking to communities, thus opening the door for municipalities to adopt stronger clean indoor air legislation. Since that time, six communities in our study sample have mobilized to adopt stronger legislation against environmental tobacco smoke, requiring all public areas (e.g., workplaces, restaurants) to be 100% smoke-free, without any exemptions (e.g., bars). Importantly, five of the 12 intervention communities in our study adopted local 100% smoke-free ordinances, compared to only one of the 12 control communities  (Χ^2^_1_ = 3.6, *P* = .06) (7). Because only 15 months have elapsed since the legislation went into effect, many communities may still be in the process of mobilizing their resources, and continued follow-up is essential for further evaluation of this trend.

The data shown here are the first to be presented that have a direct bearing on criticism of PUP law enforcement. The results suggest that pursuing an aggressive youth access agenda does not interfere with implementation of other tobacco control programming and that such pursuit may actually stimulate community-based efforts to legislate stronger anti-tobacco practices.

## Figures and Tables

**Table.** Comparison of Characteristics of Intervention and Control Communities (N = 24) in a Study on Enforcement of Laws on Youth Possession, Use, and Purchase (PUP) of Tobacco, Illinois, 2001–2005

Community	Total Population^a^	Minority, %	Latino, %	Median Household Income,^a^ $	Obtained Less Than High School Education, %	No. of PUP Law Citations Issued per Year During Study, Mean	Police Department Readiness for Enforcement at Baseline^b^
**Intervention**							
Town 1	43,000	11	3	81,000	6	11.5	3.4
Town 2	34,000	30	5	30,000	26	20.5	4.3
Town 3	9,000	4	6	47,000	19	12.8	5.3
Town 4	6,000	5	2	133,000	4	0.5	2.9
Town 5	28,000	14	6	48,000	18	52.2	2.7
Town 6	12,000	25	4	71,000	12	2.8	3.1
Town 7	20,000	7	3	45,000	22	28.0	2.3
Town 8	7,000	11	18	39,000	29	10.5	5.0
Town 9	56,000	19	5	57,000	15	12.0	2.7
Town 10	6,000	19	26	45,000	32	7.8	5.4
Town 11	22,000	55	4	60,000	19	11.8	4.9
Town 12	20,000	18	7	71,000	8	28.2	4.4
Mean	22,000	18	7	61,000	18	16.5	3.9
**Control**							
Town 1	36,000	25	28	54,000	25	17.0	2.7
Town 2	5,000	41	16	59,000	21	2.5	4.4
Town 3	7,000	3	4	72,000	15	9.0	4.1
Town 4	14,000	11	6	44,000	22	3.0	4.3
Town 5	25,000	14	6	47,000	18	6.5	3.0
Town 6	10,000	6	2	83,000	11	0.5	3.3
Town 7	7,000	50	5	57,000	15	0.0	3.4
Town 8	15,000	3	3	75,000	10	8.8	5.0
Town 9	10,000	3	4	37,000	19	4.0	3.8
Town 10	6,000	18	22	58,000	29	3.0	5.0
Town 11	26,000	26	31	59,000	27	2.8	4.6
Town 12	75,000	21	5	61,000	10	18.8	2.7
Mean	20,000	18	11	59,000	19	6.3	3.9

a Values for population and income have been rounded to the nearest thousand.

b Measure of police department's level of organizational readiness to carry out tasks related to enforcing PUP laws, scaled from 1 to 9, with 9 representing the greatest level of readiness (6).
